# Mechanism of selenomethionine inhibiting of PDCoV replication in LLC-PK1 cells based on STAT3/miR-125b-5p-1/HK2 signaling

**DOI:** 10.3389/fimmu.2022.952852

**Published:** 2022-08-18

**Authors:** Zhihua Ren, Ting Ding, Hongyi He, Zhanyong Wei, Riyi Shi, Junliang Deng

**Affiliations:** ^1^ College of Veterinary Medicine, Henan Agricultural University, Zhengzhou, China; ^2^ Key Laboratory of Animal Disease and Human Health of Sichuan Province, College of Veterinary Medicine, Sichuan Agricultural University, Chengdu, China; ^3^ Department of Basic Medical Sciences, College of Veterinary Medicine, Weldon School of Biomedical Engineering, Purdue University, West Lafayette, IN, United States

**Keywords:** PDCoV, SeMet, STAT3, miR-125b-5p-1, HK2

## Abstract

There are no licensed therapeutics or vaccines available against porcine delta coronavirus (PDCoV) to eliminate its potential for congenital disease. In the absence of effective treatments, it has led to significant economic losses in the swine industry worldwide. Similar to the current coronavirus disease 2019 (COVID-19) pandemic, PDCoV is trans-species transmissible and there is still a large desert for scientific exploration. We have reported that selenomethionine (SeMet) has potent antiviral activity against PDCoV. Here, we systematically investigated the endogenous immune mechanism of SeMet and found that STAT3/miR-125b-5p-1/HK2 signalling is essential for the exertion of SeMet anti-PDCoV replication function. Meanwhile, HK2, a key rate-limiting enzyme of the glycolytic pathway, was able to control PDCoV replication in LLC-PK1 cells, suggesting a strategy for viruses to evade innate immunity using glucose metabolism pathways. Overall, based on the ability of selenomethionine to control PDCoV infection and transmission, we provide a molecular basis for the development of new therapeutic approaches.

## Introduction

PDCoV is a novel porcine enteropathogenic coronavirus that infects intestinal epithelial cells and causes vomiting, diarrhea, dehydration and death in sows and piglets (1–3). It was first detected in Hong Kong in 2012 and its infection has subsequently been reported worldwide, posing a considerable threat to the global pig industry (4–12). In addition, recent reports have shown that chickens and calves are also susceptible to PDCoV (13, 14), and even humans are susceptible (15), indicating that the novel virus has the possibility of cross-species transmission. However, there is no specific treatment for PDCoV infection, and prevention and treatment of PDCoV infection is an urgent problem.

Selenium is an essential trace element for plants and mammals (16). Generally speaking, selenium is classified into two types: inorganic and organic selenium. Compared to inorganic selenium, organic selenium is the most valuable type of selenium intake because it is more biologically active and less toxic than inorganic selenium, as well as being more readily absorbed and utilized by living organisms and abundant in variety (17). Selenomethionine (SeMet), the main chemical form of organic selenium in cereal diets, acts as a selenium-containing amino acid by itself or as a selenium-derived donor involved in selenoprotein synthesis for its antioxidant, immunomodulatory and antiviral effects (18–23). Previous studies found that knockdown of glutathione peroxidase 1 (GPx1) in selenoprotein promoted PCV2 replication and reversed the ability of SeMet to block hydrogen peroxide (H_2_O_2_)-induced PCV2 replication (20). Furthermore, in addition to these functions, selenium also affects the metabolic levels of the body. In mouse models, mice with either selenoprotein deficiency or high selenium levels exhibit dysregulated glucose homeostasis as well as insulin resistance (24, 25).

STAT3 was originally identified as an acute phase response factor and is a member of the signal transduction and transcriptional activator family (26, 27). At the transcriptional level, it is well known for regulating the transcription of target genes for various biologically important functions, such as energy metabolism, cell differentiation and immune response (28). Abnormal STAT3 function is commonly associated with disease development including viral development. For example, the X protein of HBV induces STAT3 phosphorylation (Y705) *via* JAK1 and downregulates the expression of miRNA let-7a, a negative regulator of STAT3, thereby promoting p-STAT3 dimer-specific binding to the core structural domain of HBV enhancer 1 and inducing viral gene expression (29–31).

miRNAs consist of a class evolutionarily conserved, endogenous non-coding small RNAs of approximately 9-24 nt in length (32). miRNAs function as post-transcriptional regulators of gene expression by acting on complementary target sequences in the mRNA-3’UTR to cleave mRNAs or repress protein translation (33). miRNAs redefine the ability of host-virus interactions is an emerging concept. For example, miR-144 can target and inhibit TRAF6 levels, impairing IRF7-mediated antiviral signalling and leading to dysregulation of antiviral gene expression in the host, thereby enhancing influenza A virus (IAV) replication (34).

Host cells possess the energy and molecular precursors required for viral infection and are the ‘viral factories’ that supply the resources for processing. It has been shown that HK2, a key rate-limiting enzyme of glycolysis, is involved in the activation of retinoic acid-inducible gene-I like receptors (RLRs) mediated innate immune signalling (35), and that hepatitis B virus can use HK2 to inhibit RLRs signalling and thus achieve immune escape (36). It is from the perspective of HK2 regulation of innate immune signaling that our experiment explores the molecular mechanism of SeMet inhibiting PDCoV replication. Our results show that HK2 promotes PDCoV replication in LLC-PK1 cells, while SeMet inhibits PDCoV replication by reducing HK2 expression. Further analysis at the transcriptional level revealed that STAT3/miR-125b-5p-1 is an intermediate link in the regulation of HK2 levels by SeMet and a key mechanism for SeMet inhibiting PDCoV replication.

## Materials and methods

### Reagents and antibodies

Antibodies for NF-κB p65 (#6956), p-NF-κB p65 (#3033) and β-Actin (#4970) (Cell Signaling Technology), p-TBK1 (#AF8190) and p-STAT3 (#AF3293) (Affinity Biosciences), STAT3 (#GB12176, Servicebio), HK2 (#22029-1-AP) and IRF3 (#11312-1-AP) (Proteintech Group), p-IRF3 (#MA5-14947, Invitrogen), TBK1 (#ab227182), Goat Anti-Rabbit IgG H&L (HRP) (#ab6721) and Rabbit Anti-Mouse IgG H&L(HRP) (#ab6728) (Abcam) were used for blotting. The anti-PDCoV-N antibodies were prepared in our laboratory. The Alexa Fluor 488-conjugated goat anti-mouse (#4408S) was purchased from Cell Signaling Technology. The STAT3 inhibitor static (#HY-13818) was purchased from Med Chem Express. The selenomethionine (SeMet) (#S3132) was purchased from Sigma. The miR-9-5p mimic, miR-125b-5p-1 mimic, and miR-125b-5p-1 inhibitor were purchased from Gene Pharma.

### Cell culture and virus

The PDCoV strain-HNZK-04 (GenBank accession number MH708124) was isolated and identified by the laboratory and propagated in porcine kidney proximal tubular epithelial cell line (LLC-PK1, ATCC CL-101). LLC-PK1 cells were cultured at 37°C in 5% CO_2_ minimum essential medium (MEM, Solarbio) containing 8% fetal bovine serum (FBS, Gibco), 1% HEPES (Solarbio) and 1% MEM-nonessential amino acids (NEAA, Solarbio). Passage 34 (P34) of PDCoV HNZK-04 was cultured in MEM supplemented with 1% HEPES and 3 μg/mL trypsin (Sigma).

### Plasmids construction and transformation

The sequence of pcDNA3.1(+)-EGFP/RFP containing cloned HK2 gene (NM_001122987.1) or STAT3 gene (NM_001044580.1) were designed and ordered from the Wuhan Gene Creat, China. The pcDNA3.1-HK2/STAT3 include Neomycin resistance gene as a known selectable marker of stable mammalian transfectants and β-lactamase (the Ampicillin resistance gene) as the selectable marker in the properly transformed prokaryotic hosts, and eucaryote expression vector pcDNA3.1 (+) and target products were digested with NheI and EcoRI. TOP10 competent cells were thawed on ice, then plasmid DNA was added, mixed gently, and the mixture incubated on ice for 30 min. Cells were then transferred to a 42°C water bath for 90 sec, then placed on ice for a further 2 min. Sterile SOC liquid medium (200 µL, Solarbio) without antibiotic was added, and the mixture was incubated for 45 min at 37°C in a shaker at 220 rpm/min to recover the cells. Cells were plated on solid LB containing agar and 50 mg/mL ampicillin (Sangon Biotech) and incubated at 37°C for 12–16 h. Single colonies were then picked into 100 mL LB and incubated at 37°C for 16 h in a shaker at 220 rpm/min.

### siRNA-mediated silencing

LLC-PK1 cells [2×10 (5)] were seeded in 24-well plate for each well and allowed to grow for 12 hours. Then, cells were transfected with transfection reagent Lipofectamine 3000 (Invitrogen, #L3000015) and siRNA (HK2 and STAT3) or scrambled siRNA (siRNA-negative control, NC), Lipofectamine 3000: 3μL, 20μmol/L siRNA: 1.5μL for each well for 24 hours. The siRNA sequences are shown in **Supplementary Table S1**.

### Quantitative real-time PCR

Total RNA of cells was extracted using Trizol reagent (Takara), according to the manufacturer’s protocol. After reverse transcription by using TransScript All-in-One First-Strand cDNA Synthesis SuperMix for qPCR kit (Transgen) or Hairpin-it microRNAs RT reagent Kit (GenePharma), cDNAs were quantified in CFX Connect Real-Time PCR Detection System (BIO-RAD). Relative gene expression levels were calculated using the formula 2^-(ΔΔCt)^ (with β-actin or U6 used as the reference gene) and normalized as indicated. The information of primers are all listed in **Supplementary Table S1**.

### Luciferase reporter assays

Luciferase constructs were generated by cloning porcine 3’UTR of HK2 (NM_001122987, 2941-3118 nt), HK2 promotor (NC_010445, -967 to -768 bp relative to the transcription start site), or miR-125b-1 promotor (NC_010451, -1059 to -1258 bp relative to the transcription start site) of firefly luciferase. Wild type and mutant miR-125b-5p-1 target sequences in the 3’UTR of HK2 were generated by containing predicted interaction sites, and cloning directly into the pmirGLO vectors. Similarly, wild type and mutant STAT3 target sequences in the HK2 or miR-125b-1 promotors were cloned into the pGL3 vectors. The cells were collected 48 hours after co-transfected with pmirGLO-HK2 and miR-125b mimic, pcDNA3.1-STAT3 and pGL3-HK2, as well as pcDNA3.1-STAT3 and pGL3-miR-125b-1 using Lipofectamine 2000 (Invitrogen). The luciferase activity was measured using the Dual-Luciferase Reporter Assay System (Promega). The results are expressed as relative luciferase activity (firefly luciferase/renilla luciferase).

### Western immunoblotting

For the western blotting of the total lysates, we lysed cells in RIPA buffer (Servicebio) supplemented with phosphatase and protease inhibitors (Servicebio), which in turn subjected the lysates to protein quantification (Beyotime). The lysates were immunoblotted, electrophoresed on SDS-PAGE, transferred to PVDF membranes (Millipore), and probed with various antibodies. Chemiluminescent signals were detected by ChemiScope 6300 Touch (CLINX). Protein quantification was performed by Image J (NIH).

### Immunofluorescence assay

Cells were fixed with 4% paraformaldehyde at room temperature for 20 minutes and washed with PBS three times. Cells were permeated with 0.05% Triton-x100 for 20 min and then washed with PBS three times. Cells were blocked (5% BSA sealing fluid) for 30 min and incubated with anti- PDCoV- N antibody for 12 hours at 4°C. Cells were then washed with PBS three times and incubated with FITC- conjugated secondary antibody for 30 min at 37°C. Thereafter, cells were incubated with 4′,6- diamidino- 2- phenylindole (DAPI, Solarbio) for 10 minutes after washing with PBS three times. The cells were examined using fluorescence microscope (Nikon).

### Statistical analysis

All of the statistical data are presented as the mean ± s.e.m. Differences between mean values of normally distributed data were assessed by the one-way ANOVA test and Student’s *t*-test. Statistical difference was accepted at *P* < 0.05. The software used are Excel (Microsoft Corporation), SPSS 25.0 software (SPSS Inc.), and Prism 8.0 (GraphPad Software Inc.).

## Results

### SeMet inhibits the infectivity of PDCoV

**Figure 1:**
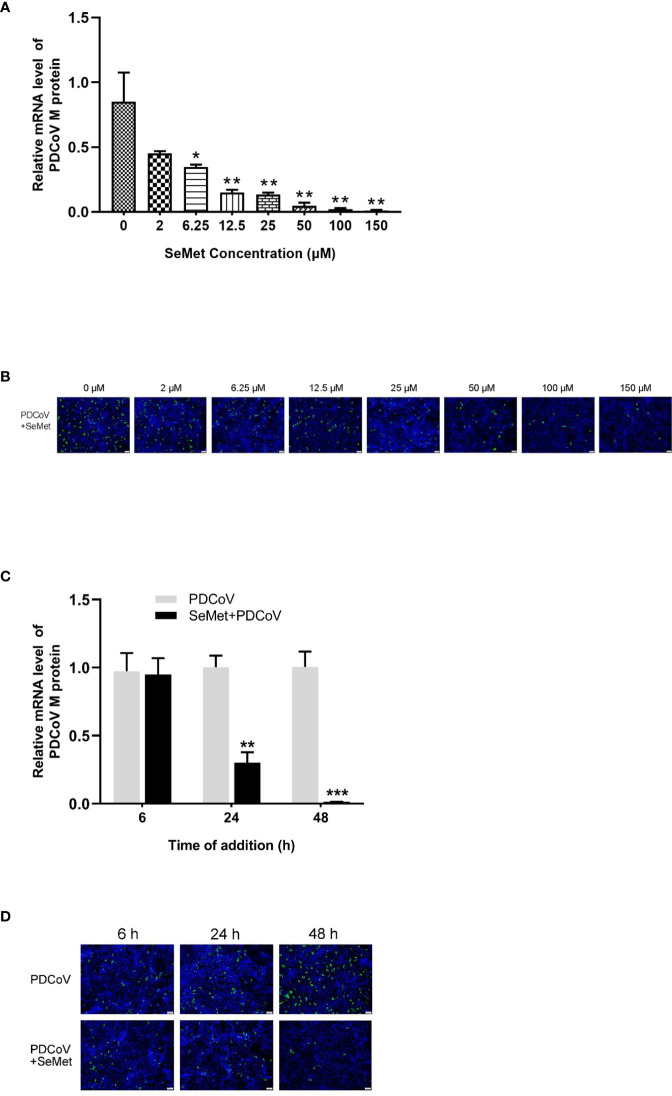
SeMet inhibits the infectivity of PDCoV. **(A)** LLC-PK1 were infected with PDCoV (100 TCID_50 =_ 10^-2.15^) for 1.5 h and treated with 0~150 μM of SeMet. Cells were collected after 48 h for qRT-PCR of viral M gene (n=6). **(B)** At 48 h treatment with SeMet, PDCoV (100 TCID_50_) replication in LLC-PK1 cells was determined by indirect immunofluorescence assay (IFA). **(C)** LLC-PK1 cells were infected with PDCoV for 1.5 h and treated with SeMet (150 μM) for 6, 24, and 48 (h) Expression level of viral mRNAs was analyzed by qRT-PCR (n=6). **(D)** At 6~48 h treatment with SeMet, PDCoV replication in LLC-PK1 cells was determined by IFA. Means ± SD are shown. Statistical significance was determined by Student *t* test. ***, *P*<0.001; **, *P*<0.01; *, n.s, not significant. All experiments were repeated at least twice and representative results are shown.

Previous assays obtained that no differences in cell morphology compared with control cells were observed cells at the maximum safe concentration with 256 μM of SeMet (data not shown). To evaluate whether SeMet inhibits PDCoV replication, LLC-PK1 cells were infected with PDCoV at 100 TCID_50_ for 1.5 h and treated with 2-256 μM of SeMet for 48 h. The relative mRNA expression was detected by real-time quantitative PCR (qPCR). Treatment with 6.25-150 μM SeMet inhibited viral mRNA levels (**Figure 1A**), while treatment with 200-256 μM SeMet infected with PDCoV for 48 h resulted in almost no viable cells. IFA assays showed that the fluorescence signal was reduced in a dose-dependent manner following treatment of PDCOV-infected LLC-PK1 cells with 6.25-150 μM SeMet (**Figure 1B**). This indicated that SeMet significantly inhibited PDCoV replication in a dose-dependent manner, and we selected 150μM SeMet for further studies in subsequent assays.

To further elucidate the effect of SeMet on PDCoV, we performed analyses of SeMet treatment at different times. 6, 24 and 48 h after PDCoV infection were treated with SeMet. we observed a decrease in viral mRNA of PDCoV with increasing time of infection (**Figure 1C**), as well as a weakening of the IFA detection fluorescence signal (**Figure 1D**). This suggests that SeMet significantly inhibited PDCoV replication in a time-dependent manner.

### HK2 plays an important role in PDCoV replication

**Figure 2:**
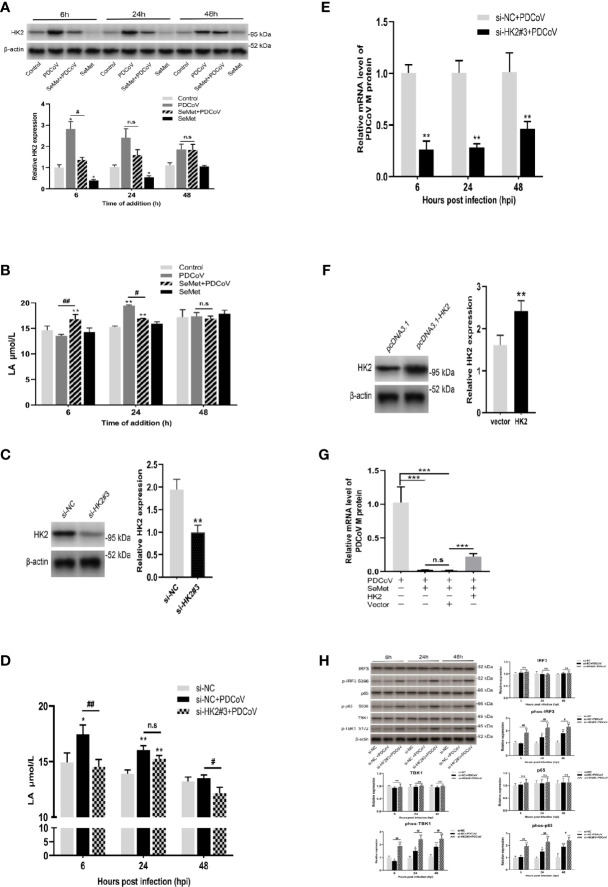
HK2 plays an important role in PDCoV replication. **(A)** LLC-PK1 cells were treated with PDCoV (100 TCID_50_) and SeMet (150 μM) for 6, 24, and 48 h, and the level of HK2 was determined by and Western blot(n=3). **(B)** The LA level was measured by ELISA (n=3). **(C)** The HK2 protein expression was determined by Western blot in LLC-PK1 cells treated with siRNA-NC and siHK2 (n=3). **(D)** HK2 inhibition-induced LA expression after PDCoV infection. LA expression in LLC-PK1 cells was determined by ELISA (n=4). **(E)** Cells were infected with PDCoV for 6, 24, and 48 h post infection (hpi), after transfection with siRNA-NC or siHK2. Expression level of viral mRNAs was analyzed by qRT-PCR (n=8). **(F)** The HK2 protein expression was determined by Western blot in LLC-PK1 cells when transfected with pcDNA3.1-vector or pcDNA3.1-HK2 (n=3). **(G)** Cells were transfected with pcDNA3.1-vector or pcDNA3.1-HK2, followed by treatment with SeMet and PDCoV. Viral mRNA expression was analyzed by qRT-PCR (n=6). **(H)** HK2 inhibition-induced RLR signal proteins expression after PDCoV infection. The TBK1, phos-TBK1 (Ser172), IRF3, phos-IRF3 (Ser396), p65, or phos-p65 (Ser536) protein levels were analyzed by Western blot (n=3). Means ± SD are shown. Statistical significance was determined by Student *t* test. ***, *P*<0.001; **, *P*<0.01; *, *P*<0.05; ^##^, *P*<0.01; ^#^, *P*<0.05; n.s: not significant. All experiments were repeated at least twice and representative results are shown.

We next explored how SeMet affects PDCoV replication. Our study found that SeMet treatment of cells infected with PDCoV resulted in a reduction in HK2 and lactate expression (**Figures 2A, B**). Previous studies have shown that lactate is critical for blocking RLRs signalling associated with viral infection, and that HK2 can influence the activation of RLRs signalling by regulating lactate production (35). We hypothesized that this role is precisely exploited by SeMet. Therefore, we knocked down the expression of HK2 (**Figure 2C** and **Supplementary Figures S1A, B**). We found that inhibition of HK2 expression attenuated the PDCoV-induced elevation of lactate (**Figure 2D**). Also, inhibition of HK2 expression reduced PDCoV replication at various times (**Figure 2E**), while restoration of HK2 expression attenuated the ability of SeMet inhibiting PDCoV replication (**Figures 2F, G** and **Supplementary Figures S1C, D**). These data suggest that SeMet inhibits PDCoV replication by reducing HK2 levels.

Considerable research has shown that PDCoV can achieve immune escape by inhibiting the activation of RLRs signalling (37–41). To further investigate the role of HK2 on the RLRs signalling pathway under the influence of PDCoV infection, we also examined the effect of HK2 inhibition on the total protein and phosphorylation of TBK1, IRF3 and p65, key proteins in RLRs signalling. We observed no significant changes in total protein expression in the three groups (**Figure 2H**), whereas knockdown of HK2 resulted in a significant increase in phosphorylation of TBK1, IRF3 and p65 in cells infected with PDCoV at different times (**Figure 2H**), suggesting that it promotes the expression of downstream signalling molecules in RLRs.

### miR-125b-5p-1 is required for HK2-dependent SeMet inhibiting PDCoV replication

**Figure 3:**
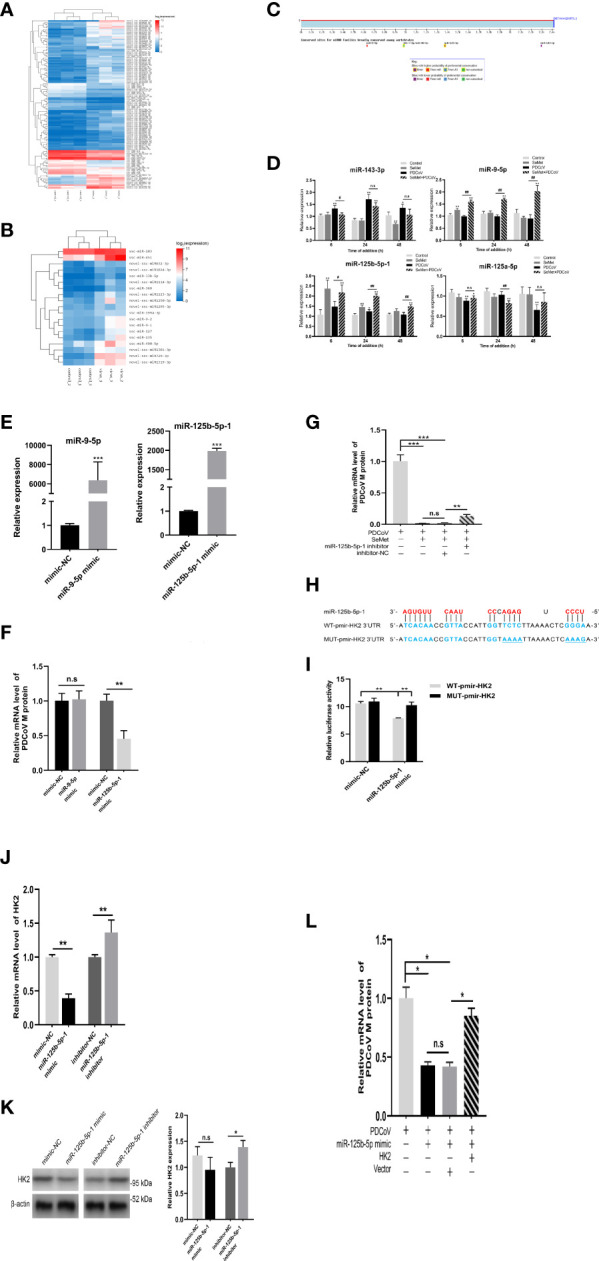
miR-125b-5p-1 is required for HK2-dependent SeMet inhibiting PDCoV replication. **(A, B)** Hierarchic clustering analyses of 118 miRNAs that were differentially expressed in the SeMet-supplemented LLC-PK1 cells by >2-fold compared with the mock-treated LLC-PK1 cells, and 19 miRNAs that were differentially expressed in the PDCoV-infected cells by >2-fold compared with the mock-treated cells. **(C)** Analysis of the TargetScanHuman (http://www.targetscan.org/vert_72/) website shows that miR-143-3p, miR-9-5p, miR-125b-5p-1, and miR-125a-5p can target the human 3’UTR of HK2. Binding sequences are highly conserved among species (including human and swine). **(D)** LLC-PK1 cells were treated with PDCoV (100 TCID_50_) and SeMet (150 μM) for 6, 24, and 48 h, and the levels of miR-143-3p, miR-9-5p, miR-125b-5p-1, and miR-125a-5p were determined by qRT-PCR (n=6). **(E)** The miR-9-5p and miR-125b-5p-1 expressions were determined by qRT-PCR when transfected with mimic-NC, miR-9-5p mimic, and miR-125b-5p-1 mimic (n=4). **(F)** Viral mRNA in the LLC-PK1 cells transfected with miR-9-5p mimic or miR-125b-5p-1 mimic was quantified by qRT-PCR after 48 h (n=6-8). **(G)** Cells were transfected with miR-125b-5p-1 inhibitor, followed by treatment with SeMet and PDCoV. Viral mRNA expression was analyzed by qRT-PCR (n=6). **(H)** The miR-125b-5p-1 targets the base sequence and mutant sequence of the 3’UTR of HK2. **(I)** The miR-125b-5p-1 mimic or mimic-NC and WT-pmirGLO-HK2 or MUT-pmirGLO-HK2 were co-transfected into HEK293T cells. The luciferase reporter assay was used to detect whether miR-125b-5p-1 targeted to bind to the 3’UTR of HK2 (n=5). **(J, K)** The HK2 mRNA and protein expressions were determined by qRT-PCR and Western blot in LLC-PK1 cells when transfected with miR-125b-5p-1 mimic or inhibitor (n=3-6). **(L)** LLC-PK1 cells were transfected with miR-125b-5p-1 mimic and pcDNA3.1-HK2, followed by infection with PDCoV. Viral mRNA expression was analyzed by qRT-PCR (n=6). Means ± SD are shown. Statistical significiance was determined by Student *t* test. ***, *P*<0.001; **, *P*<0.01; *, *P*<0.05; ^##^, *P*<0.01; ^#^, *P*<0.05; n.s: not significant. All experiments were repeated at least twice and representative results are shown.

To elucidate the mechanism whereby SeMet inhibits PDCoV replication within LLC-PK1 cells *via* HK2, we compared the miRNA transcriptional profiles of PDCoV-infected or SeMet-supplemented cells with those of blank control cells, respectively. SeMet supplementation significantly increased the expression of 84 miRNAs and decreased the expression of 34 miRNAs in LLC-PK1 cells; while PDCoV infection upregulated the expression of 19 miRNAs (**Figures 3A, B**). Combining RNA-seq and TargetScan database predictions of miRNAs targeting the HK2 gene, we validated the differential expression of several characteristic and possible antiviral miRNAs (miR-143-3p, miR-9-5p, miR-125b-5p-1 and miR-125a-5p) by qRT-PCR (**Figure 3C**). We found that the addition of SeMet caused elevated expression of miR-9-5p and miR-125b-5p-1 at different time points (**Figure 3D**). So does SeMet inhibit PDCoV replication by increasing the expression of miR-9-5p and miR-125b-5p-1? Next, we synthesized miR-9-5p mimic and miR-125b-5p-1 mimic to increase their expression levels in LLC-PK1 cells (**Figure 3E** and **Supplementary Figure S1E**). Overexpression of miR-9-5p had no effect on PDCoV replication; whereas overexpression of miR-125b-5p-1 significantly inhibited PDCoV replication (**Figure 3F**). We further verified whether the antiviral effect of SeMet was dependent on the regulation of miR-125b-5p-1 and found that down-regulation of miR-125b-5p-1 expression attenuated the ability of SeMet to inhibit PDCoV replication (**Figure 3G** and **Supplementary Figures S1F, G**). These results suggest that miR-125b-5p-1 can negatively regulate PDCoV replication in LLC-PK1 cells and that SeMet inhibition of PDCoV replication is dependent on the positive regulation of miR-125b-5p-1.

Meanwhile, to determine whether HK2 is a direct target gene of miR-125b-5p-1, we constructed the porcine 3’UTR of HK2 luciferase reporter plasmid (**Figure 3H**). Reporter activity analysis showed that miR-125b-5p-1 mimic reduced the luciferase activity of the reporter containing 3’UTR of HK2 in HEK293T cells but had no effect on the reporters with mutated 3’UTR of HK2 (**Figure 3I**), suggesting that HK2 is indeed a miR-125b-5p -1 target. We also demonstrated using qRT-PCR and Western blot that overexpression of miR-125b-5p-1 suppressed HK2 expression and knockdown of miR-125b-5p-1 promoted HK2 transcription and protein expression (**Figures 3J, K**). To further determine the effect of miR-125b-5p-1/HK2 on PDCoV infection, we performed a rescue experiment. Co-transfection of miR-125b-5p-1 mimic and HK2 overexpression plasmid showed that restoration of HK2 expression attenuated effects of overexpression of miR-125b-5p-1 in inhibiting PDCoV replication (**Figure 3L**). These data suggest that miR-125b-5p-1 inhibition of PDCoV replication in LLC-PK1 cells is dependent on the negative regulation of the HK2 gene.

### STAT3 directly regulates miR-125b-5p-1 expression

**Figure 4:**
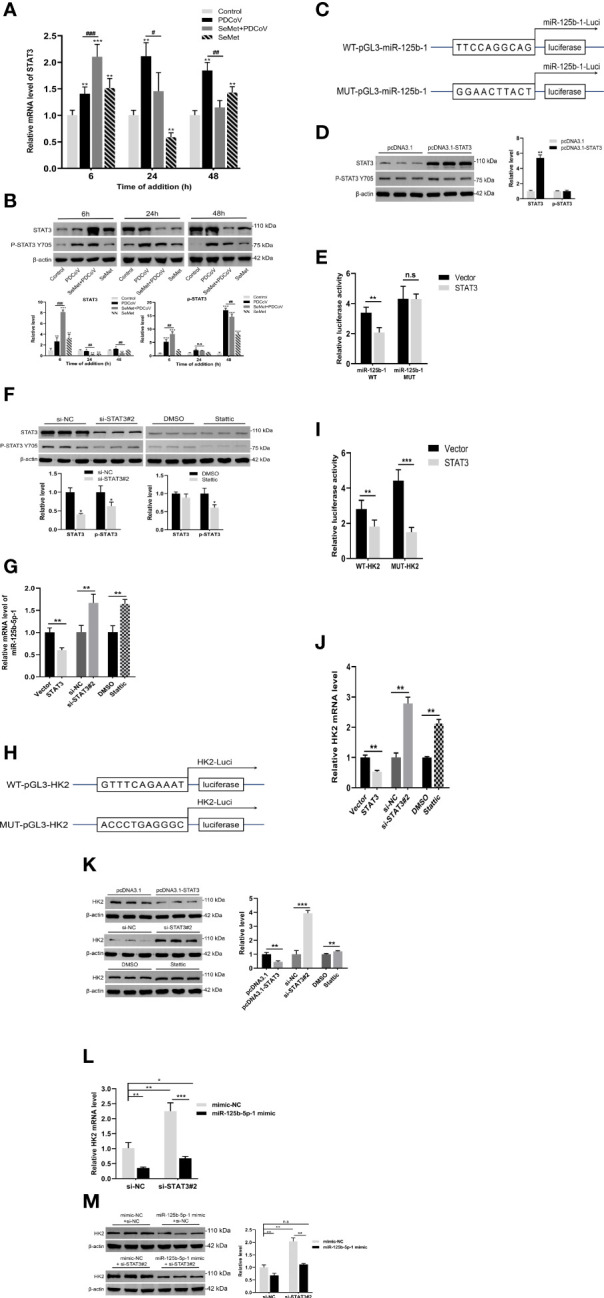
STAT3 directly regulates miR-125b-5p-1 expression. **(A, B)** LLC-PK1 cells were treated with PDCoV (100 TCID_50_) and SeMet (150 μM) for 6, 24, and 48 h, and the level of STAT3 and phos-STAT3 (Tyr705) was determined by qRT-PCR or Western blot (n=3-6). **(C)** The base sequence and mutant sequence of miR-125b-5p-1 promoter. **(D)** The STAT3 protein expressions was determined by qRT-PCR and Western blot in LLC-PK1 cells when transfected with pcDNA3.1-vector or pcDNA3.1-STAT3 (n=3). **(E)** The pcDNA3.1-STAT3 or empty vector and WT-pGL3-miR-125b-1 or MUT- pGL3-miR-125b-1 were co-transfected into HEK293T cells. The luciferase reporter assay was used to detect whether STAT3 can target and bind on miR-125b-1 promoter (n=5). **(F)** The STAT3 protein expression was determined by qRT-PCR or Western blot in LLC-PK1 cells treated with siRNA-NC, siSTAT3, DMSO, and Stattic (STAT3 inhibitor, 0.75 μM) (n=3). **(G)** The miR-125b-5p-1 expression was measured by qRT-PCR when transfected with pcDNA3.1-STAT3, empty vector, siSTAT3, siRNA-NC, DMSO, Stattic (0.75 μM) (n=6). **(H)** The base sequence and mutant sequence of HK2 promoter. **(I)** The pcDNA3.1-STAT3 or empty vector and WT-pGL3-HK2 or MUT- pGL3-HK2 were co-transfected into HEK293T cells. The luciferase reporter assay was used to detect whether STAT3 can target and bind on HK2 promoter (n=5). **(J, K)** The HK2 mRNA and protein expressions were determined by qRT-PCR and Western blot in LLC-PK1 cells when transfected with pcDNA3.1-STAT3, empty vector, siSTAT3, siRNA-NC, DMSO, Stattic (0.75 μM) (n=3-6). **(L, M)** The siSTAT3 or siRNA-NC and miR-125b-5p-1 mimic or mimic-NC were co-transfected into LLC-PK1 cells. The HK2 mRNA and protein expressions were determined by qRT-PCR and Western blot (n=3-6). Means ± SD are shown. Statistical significiance was determined by Student *t* test. ***, *P*<0.001; **, *P*<0.01; *, *P*<0.05; ^###^, *P* < 0.001; ^##^, *P*<0.01; ^#^, *P*<0.05; n.s: not significant. All experiments were repeated at least twice and representative results are shown.

We found that in LLC-PK1 cells infected with PDCoV, SeMet treatment resulted in a significant increase in STAT3 expression levels in the early stages and a decrease in the middle and late stages (**Figures 4A, B**). Next, we further analyzed the miR-125b-1 promoter sequence and identified interaction sites at -1059 to -1258 of the miR-125b-1 transcriptional start, which are STAT3 binding sites. We constructed a luciferase reporter plasmid containing the miR-125b-1 promoter (**Figure 4C**) and a STAT3 overexpression plasmid vector (**Figure 4D** and **Supplementary Figures S1H, I**). The pcDNA-STAT3 plasmid or empty vector was cotransfected with WT-pGL3-miR-125b-1 plasmid or mutant plasmid. The results of reporter gene activity analysis showed that STAT3 reduced luciferase activity in the miR-125b-1 promoter, but had no effect on the reporter gene mutated in the miR-125b-1 promoter (**Figure 4E**), suggesting that miR-125b-5p-1 is indeed a target of STAT3. The qRT-PCR results also showed that overexpression of STAT3 reduced the activity of miR-125b-5p-1 expression levels, while inhibition of STAT3 levels increased miR-125b-5p-1 expression (**Figures 4F, G** and **Supplementary Figures S1J, K**). These data suggest that STAT3 directly regulates the expression of miR-125b-5p-1.

Similarly, we identified interaction sites with STAT3 at -967 to -768 of the HK2 transcription start site and constructed a luciferase reporter plasmid containing the HK2 promoter (**Figure 4H**). Analysis of reporter gene activity showed that STAT3 reduced the luciferase activity of the HK2 promoter and that the luciferase activity of the mutant was also reduced (**Figure 4I**). We also demonstrated that overexpression of STAT3 inhibited HK2 transcription and protein expression and that inhibition of STAT3 expression promoted HK2 transcription and protein levels (**Figures 4J, K**). These data suggest that the -967 to -768 sites in the HK2 promoter sequence are not targets of STAT3 and that other interaction sites or potential regulators may exist.

In our study, we found that miR-125b-5p-1 could target and inhibit the expression of HK2, while STAT3 could also inhibit the expression of HK2. Interestingly, we also identified a binding site for STAT3 on the promoter of miR-125b-5p-1 and demonstrated that STAT3 can repress miR-125b-5p-1 expression. These data suggest a possible feedback regulatory mechanism for STAT3/miR-125b-5p-1/HK2 to maintain intracellular glucose metabolism homeostasis. To further explore the effect on HK2 in the presence of STAT3 and miR-125b-5p-1 together, we cotransfected si-STAT3#2 and miR-125b-5p-1 mimic. The qRT-PCR and Western blot results showed that transfection of si-STAT3#2 or miR-125b-5p-1 mimic alone elevated or decreased HK2 expression, respectively, whereas the cotransfected group showed no significant change in HK2 mRNA expression or protein expression compared to the control group (**Figures 4L, M**). These data suggest that miR-125b-5p-1 plays a major regulatory role on HK2 under the combined effect of STAT3 and miR-125b-5p-1. In addition, it also confirms that SeMet can indeed suppress HK2 levels by reducing STAT3 and up-regulating miR-125b-5p-1 expression, thus exerting an anti-PDCoV effect.

### Decreased STAT3 levels are utilized by SeMet to suppress PDCoV replication

**Figure 5:**
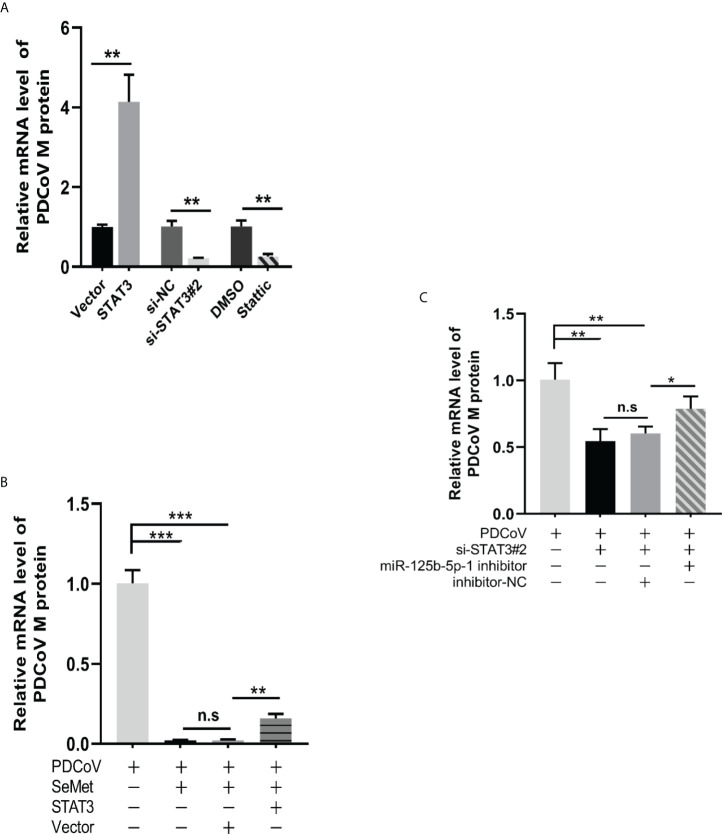
Decreased STAT3 levels are utilized by SeMet to suppress PDCoV replication. **(A)** Expression level of viral mRNAs was analyzed by qRT-PCR when transfected with pcDNA3.1-STAT3, empty vector, siSTAT3, siRNA-NC, DMSO, Stattic (0.75 μM) (n=6). **(B)** Cells were transfected with pcDNA3.1-vector or pcDNA3.1-STAT3, followed by treatment with SeMet and PDCoV. Viral mRNA expression was analyzed by qRT-PCR (n=6). **(C)** Expression level of viral mRNAs was analyzed by qRT-PCR when transfected with siSTAT3 and miR-125b-5p-1 inhibitor followed by infection with PDCoV (n=6). Means ± SD are shown. Statistical significiance was determined by Student *t* test. ***, *P*<0.001; **, *P*<0.01; *, *P*<0.05; n.s, not significant. All experiments were repeated at least twice and representative results are shown.

We further explored whether STAT3 could act as an upstream of miR-125b-5p-1/HK2 signalling to influence PDCoV replication in LLC-PK1 cells. In conjunction with the previously mentioned ability of SeMet addition to show differential changes in STAT3 expression at different time points, we therefore investigated the effects on PDCoV replication at both overexpressed and knockdown STAT3 transcript and protein levels. The si-STAT3 interference or the STAT3 inhibitor Stattic reduced the mRNA expression of PDCoV M gene, whereas pcDNA-STAT3 increased the mRNA expression of M gene (**Figure 5A**). Next, we verified the link between STAT3 and SeMet or STAT3 and miR-125b-5p-1 in affecting PDCoV infection. The data showed that restoration of STAT3 expression attenuated the ability of SeMet to resist PDCoV replication (**Figure 5B**) and inhibition of miR-125b-5p-1 mRNA expression attenuated the role of si-STAT3 in inhibiting PDCoV replication (**Figure 5C**).

## Discussion

Since 2012, when PDCoV was first identified in Hong Kong, China. There has been continued research into PDCoV vaccines, but no approved treatments or vaccines for PDCoV are currently on the market. Laboratory studies at this stage have focused on drugs to combat PDCoV infection *in vitro*. Melatonin (42), cholesterol 25 hydroxylase (CH25H) (43), goose deoxycholic acid (CDCA) and lithophanic acid (LCA) (44) have been found to have antiviral activity against a PDCoV-infected porcine kidney cell line (LLC-PK1). Our results showed that SeMet significantly inhibited the replication of PDCoV in LLC-PK1 cells, with the inhibitory effect enhanced in a concentration-dependent/time-dependent manner.

SeMet’s low toxicity, high bioavailability and its use as an effective anti-PDCoV drug has raised expectations for its clinical application. Although increasing selenium intake in selenium-sufficient individuals is not advocated, some studies have shown that body selenium levels are positively correlated with cure rates and negatively correlated with morbidity and mortality in patients with viral infections, and that high selenium levels may attenuate the deleterious effects of viral infections (45–48). In addition, numerous studies have demonstrated the important role of selenium compounds in the fight against viral infections, such as the inhibition of human herpesvirus type 1/type 2, cerebral myocarditis virus, vesicular stomatitis virus, porcine circovirus type 2, EBV and hepatitis B/C virus replication (20, 49–53), so it is feasible to increase body selenium levels during the phase of viral infection in response to the acute phase.

Here, we found that early infection of LLC-PK1 cells by PDCoV significantly increased the expression of HK2 and LA in the cells and attenuated p-TBK1, a key protein in the RLRs signalling pathway, and the activation of the RLRs signalling pathway was inhibited; whereas when the expression of HK2 was reduced, the inhibitory effect of viral infection on the activation of the RLRs signalling pathway was attenuated and the expression of p-IRF3 and p-p65 proteins was further increased and viral replication was simultaneously reduced. The implication that HK2 can influence PDCoV replication in cells and its important role in the RLRs signalling pathway is supported by recent data (35, 36). Next, we explored the molecular mechanisms by which SeMet inhibits PDCoV. Indeed, selenium is able to play an immunomodulatory or metabolic regulatory role by participating in selenoprotein synthesis as a selenium-containing amino acid itself or as a selenium-derived donor (21, 23, 54, 55). HK2, the initial key rate-limiting enzyme that catalyzes the glycolytic reaction, was found to be significantly upregulated in studies of multiple malignant diseases and was accompanied by high glucose uptake and increased rates of glycolysis (56). HK2 acts as a hub linking glycolysis and innate immunity, and we demonstrated that it plays a key role in the SeMet anti-PDCoV process.

MicroRNAs are involved in a variety of biological processes, including virus-mediated host innate immune responses, by repressing the expression of target genes. There have been few studies on miRNA and coronaviruses, but the relationship between SARS-CoV-2 infestation and miRNA regulation has been extensively studied in the last two years. Wang et al. (57) combined bioinformatics analysis and laboratory assays to screen for four inhibitory effects on spike-in glycoprotein (S protein) miRNAs (miR-223-3p, miR-24-3p, miR-145-5p, and miR-7-5p). Interestingly, the expression of CoV2-miR-7a.2 encoded by SARS-CoV-2 was comparable in infected cells to one of the most abundant human miRNAs, has-miR-let-7a, and the efficiency of CoV2-miR-7a.2 and Argonaute protein forming RNA silencing complex (degrading target mRNAs) was similar to that of miR-let-7a; in addition, CoV2-miR-7a.2 can target the human BATF2 gene (an interferon-stimulated gene) and inhibit ISG expression, thereby promoting SARS-CoV-2 replication (58). In our initial experiments, we have predicted miRNAs that can target binding to HK2 through bioinformatics databases and screened miR-125b-5p-1 for follow-up testing. The ability of miR-125b-5p to target the HK2 gene to affect cellular glycolysis has been reported to a lesser extent in recent years, but almost all studies have been related to cancer. Hui et al. (59) found that miR-125b-5p was significantly down-regulated in laryngeal carcinoma tissues and cell lines and that overexpression of miR-125b-5p inhibited LSCC cell proliferation and induced apoptosis. Further molecular studies showed that miR-125b-5p binds to the 3’UTR of HK2, and overexpression of miR-125b-5p downregulated the mRNA and protein level expression of HK2, significantly inhibiting glucose consumption and lactate production in LSCC cells. In a study to validate how miR-125b-5p is involved in bladder cancer (BCa) development, Liu et al. (60) found that miR-125b-5p exerts its inhibitory effect on BCA by targeting HK2 and inhibiting the PI3K/AKT pathway. In a study to address the resistance to cisplatin in some colon cancer patients, Shi et al. (61) found that overexpression of miR-125b-5p increased the sensitivity of colon cancer cells to cisplatin by directly targeting the 3’UTR of HK2 and inhibiting the glycolytic efficiency of the cells, while differentiation antagonist non-protein coding RNA (DANCR) can bind to the seed region of miR-125b-5p in the form of a competitive endogenous RNA (ceRNA) to reduce cisplatin sensitivity in colon cancer cells. All of these studies in cancer disease confirm that miR-125b-5p binds to the 3’UTR of HK2 and affects the efficiency of cellular glycolysis. Our experiments have also demonstrated that overexpression of miR-125b-5p-1 inhibits HK2 mRNA and protein expression by binding to the 3’UTR of the porcine HK2 gene, thus suppressing LA production in LLC-PK1 cells.

In addition, a few studies have shown that patients infected with viral hepatitis (HBV, HCV and HEV) are often accompanied by elevated serum miR-125b-5p levels and that miR-125b-5p levels correlate with viral load and severity of liver injury (62–65). Deng et al. (66) in exploring the regulation of miR-125b-5p on HBV replication at different stages of HBV transcription and assembly found that overexpression of miR-125b-5p increased HBV nucleocapsid protein formation but did not enhance HBV promoter activity or transcription, and further identified a mechanism by which miR-125b-5p stimulated HBV replication with the help of the LIN28B/miR-98 axis. Our experiments again validated that exogenous addition of porcine miR-125b-5p-1 mimic can reduce mRNA levels of PDCoV M gene, but there is no direct evidence that miR-125b-5p-1 can target the regulation of PDCoV transcription. However, whether it can play a role in regulating PDCoV replication by affecting PDCoV promoter activity needs further investigation.

Pathogen infection causes an acute phase response and is accompanied by activation of the STAT3 signalling pathway. For example, high expression of p-STAT3 was found in endothelial cells of small pulmonary veins and interstitial capillaries from COVID-19 patients (67); as well as increased expression of p-STAT3 in COVID-19-associated collapsed glomerulopathy biopsy samples (68). This is consistent with our observation that PDCoV infection, which is also a member of the coronaviridae family, is also capable of activating STAT3 phosphorylation. However, the relationship between PDCoV and the host molecule STAT3 is not well understood and whether PDCoV can specifically activate STAT3 similarly to other viruses such as HBV (29), HCV (69, 70) and HCMV (71, 72) that encode viral proteins needs further investigation. Our preliminary findings suggest that PDCoV infection persistently activates STAT3, while overexpression of STAT3 promotes PDCoV replication and inhibition of STAT3 expression attenuates PDCoV replication.

In this study, SeMet could inhibit PDCoV replication by regulating STAT3/miR-125b-5p-1/HK2, which seems to imply that SeMet could be a potential drug for the treatment of PDCoV infection. We also found that miR-125b-5p-1 could target HK2 to inhibit PDCoV replication; while STAT3 could target the miR-125b-5p-1 promoter while non-targeting the HK2 promoter, inducing a potential loop by reducing their expression. We hypothesize that there are also regulatory factors between STAT3 and HK2 that allow STAT3/miR-125b-5p-1/HK2 to form a regulatory loop that maintains intracellular glucose metabolism homeostasis. When cells are exposed to external stimuli, this dynamic balance is disrupted and STAT3 or miR-125b-5p-1 loses a mutual balance, leading to a disruption of intracellular HK2 and even glucose metabolism, making them more susceptible to invasion by viruses or other pathogenic microorganisms.

## Data availability statement

The original contributions presented in the study are included in the article/**Supplementary Material**. Further inquiries can be directed to the corresponding author.

## Author contributions

ZR, TD and HH contributed to conception and design of the study. TD and HH performed the statistical analysis. TD wrote the first draft of the manuscript. RS wrote sections of the manuscript. ZW and JD reviewed and revised the manuscript. All authors contributed to manuscript revision, read, and approved the submitted version.

## Funding

The manuscript was supported by The National Science Foundation of China (32130106 and 31402269).

## Conflict of interest

The authors declare that the research was conducted in the absence of any commercial or financial relationships that could be construed as a potential conflict of interest.

## Publisher’s note

All claims expressed in this article are solely those of the authors and do not necessarily represent those of their affiliated organizations, or those of the publisher, the editors and the reviewers. Any product that may be evaluated in this article, or claim that may be made by its manufacturer, is not guaranteed or endorsed by the publisher.
