# Supplementary material for: Mechanism of selenomethionine inhibiting of PDCoV replication in LLC-PK1 cells based on STAT3/miR-125b-5p-1/HK2 signaling
Source: Front Immunol. 2022 Aug 18;13:952852. doi: 10.3389/fimmu.2022.952852 (PMC9436478; doi:10.3389/fimmu.2022.952852)

Supplementary Figure 1. Fluorescence transfection and transfection efficiency are shown


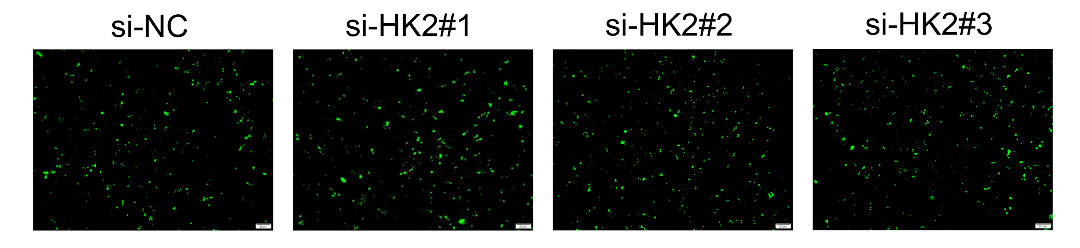


(A) Fluorescence microscopy to detect transfection efficiency of siRNA-NC and siHK2 carrying FAM fluorophore.


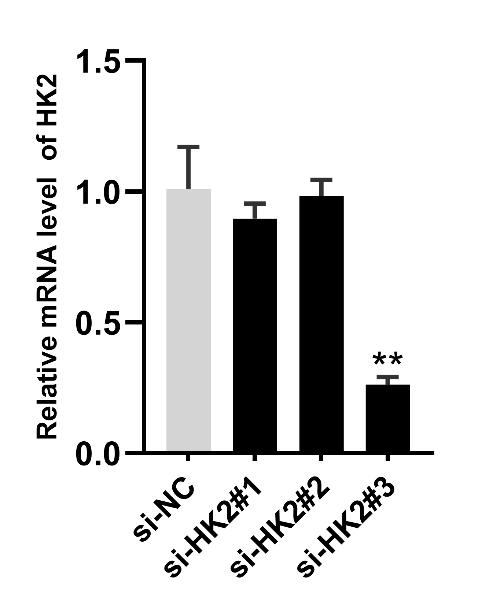


(B) The HK2 mRNA expression was determined by qRT-PCR in LLC-PK1 cells treated with siRNA-NC and siHK2 (n=6).


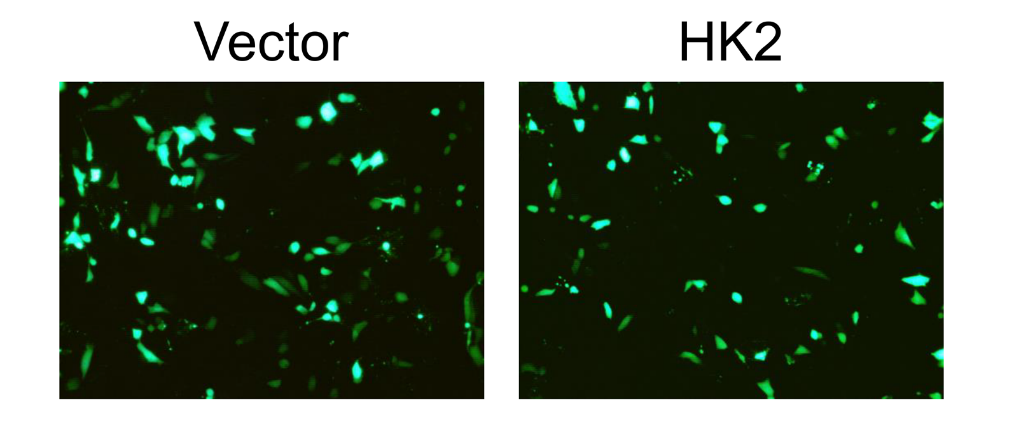


(C) Fluorescence microscopy to detect transfection efficiency of pcDNA3.1-vector or pcDNA3.1-HK2 expressing EGFP protein.


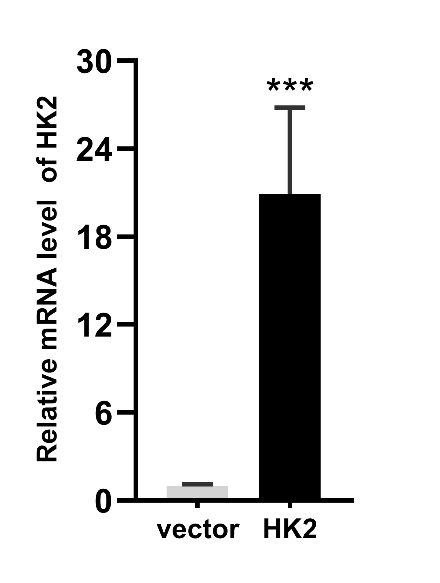


(D) The HK2 mRNA expression was determined by qRT-PCR and Western blot in LLC-PK1 cells when transfected with pcDNA3.1-vector or pcDNA3.1-HK2 (n=8).


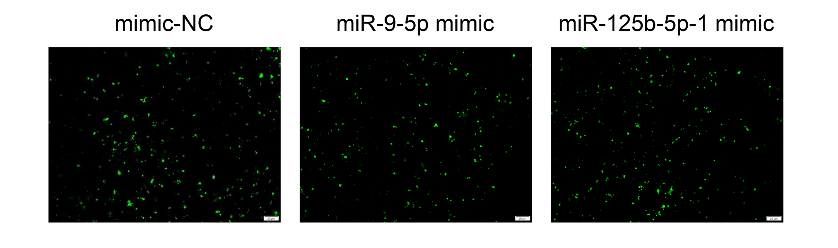


(E) Fluorescence microscopy to detect transfection efficiency of mimic-NC, miR-9-5p mimic, and miR-125b-5p-1 mimic carrying FAM fluorophore.


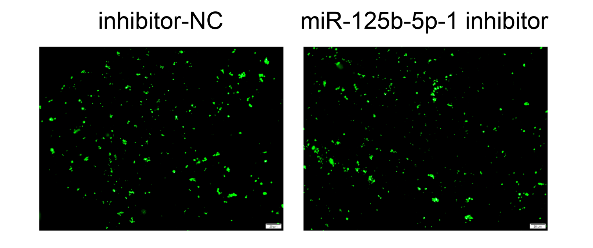


(F) Fluorescence microscopy to detect transfection efficiency of inhibitor-NC and miR-125b-5p-1 inhibitor carrying FAM fluorophore.


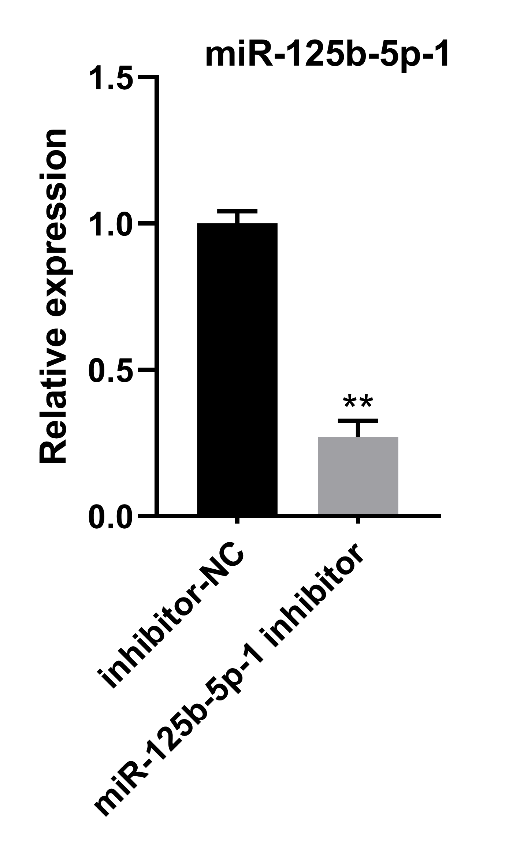


(G) The miR-125b-5p-1 expression in the LLC-PK1 cells transfected with inhibitor-NC or miR-125b-5p-1 inhibitor was quantified by qRT-PCR (n=4).


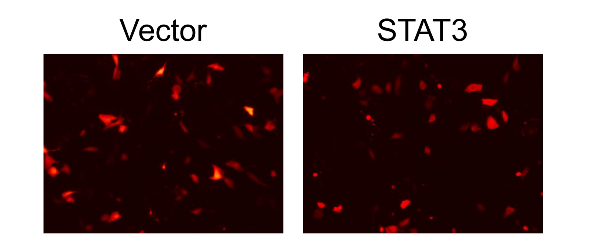


(H) Fluorescence microscopy to detect transfection efficiency of pcDNA3.1-vector or pcDNA3.1-STAT3 expressing RFP protein.


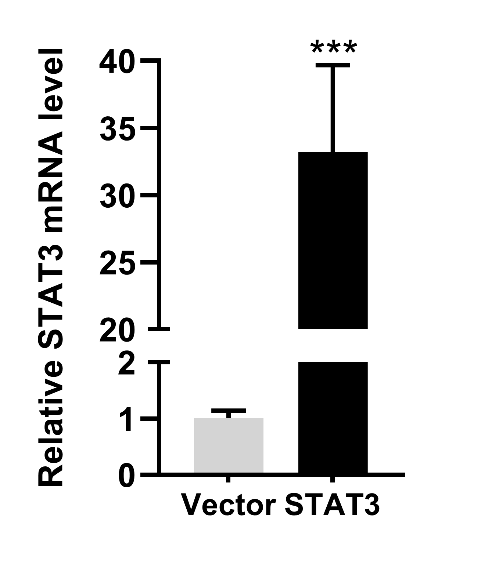


(I) The STAT3 mRNA expression was determined by qRT-PCR and Western blot in LLC-PK1 cells when transfected with pcDNA3.1-vector or pcDNA3.1-STAT3 (n=6).


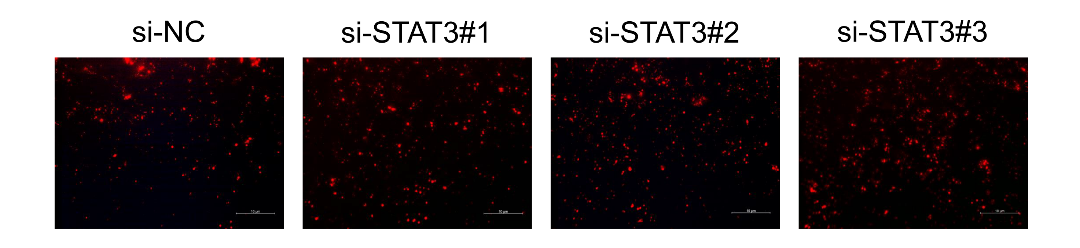


(J) Fluorescence microscopy to detect transfection efficiency of siRNA-NC and siSTAT3 carrying CY3 fluorophore.


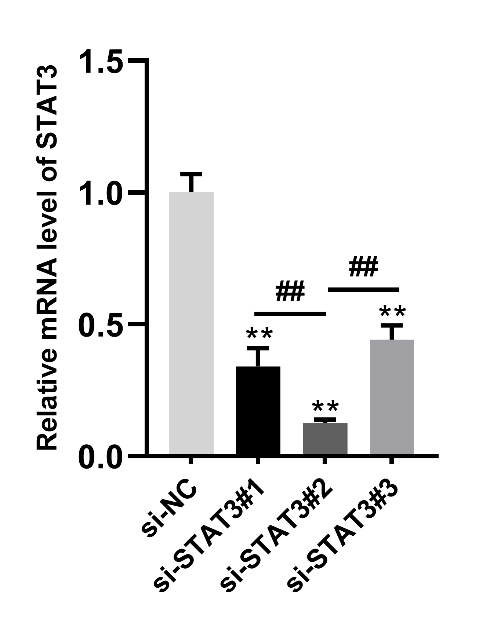


(K) The STAT3 mRNA expression was determined by qRT-PCR or Western blot in LLC-PK1 cells treated with siRNA-NC, siSTAT3, DMSO, and Stattic (STAT3 inhibitor, 0.75 μM) (n=6).

Supplementary Table 1. Oligos and primers used in the study


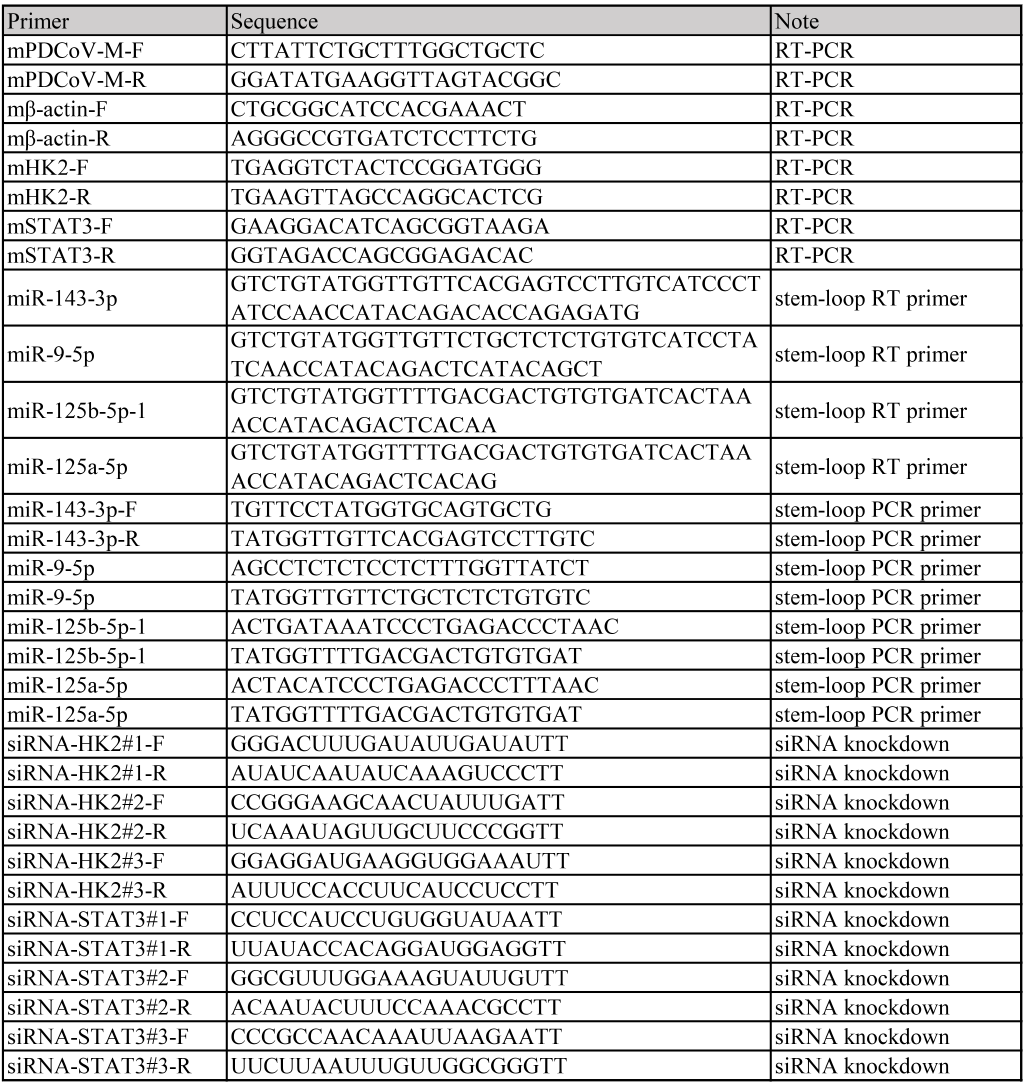

Supplement: Supplementary file 1 [file DataSheet_1.docx]
